# Discovery of a Leptospirosis Cluster Amidst a Pneumonic Plague Outbreak in a Miners’ Camp in the Democratic Republic of the Congo

**DOI:** 10.3390/ijerph110201824

**Published:** 2014-02-07

**Authors:** Eric Bertherat, Melissa J. Mueller, Jean-Christophe Shako, Mathieu Picardeau

**Affiliations:** 1 Pandemic and Epidemic Diseases, World Health Organization, Geneva 1202, Switzerland; 2 Clinical and Translational Science Institute, University of Minnesota, 717 SE Delaware Street, Minneapolis, MN 55414, USA; E-Mail: muell661@umn.edu; 3 Plague Reference Laboratory, Bunia, Democratic Republic of the Congo; E-Mail: shakochri@yahoo.fr; 4 Pasteur Institute, Biology of Spirochetes Unit, National Reference Center for Leptospirosis, WHO Collaborating Center for Leptospirosis, 75724 Paris Cedex 15, France; E-Mail: mathieu.picardeau@pasteur.fr

**Keywords:** leptospirosis, pneumonia, plague, pneumonic plague, Central Africa

## Abstract

Conditions in the Democratic Republic of the Congo provide an ideal environment for leptospirosis and plague, both of which can cause severe pulmonary manifestations. In December 2004, an outbreak of lethal pneumonia occurred in a local mining camp, affecting 130 persons and killing 57 of them. Clinical signs, fast disease spread, and initial laboratory investigations suggested pneumonic plague. While leptospirosis had not recently been described in the region, it was considered as a differential diagnosis. Anti-*Leptospira* antibodies were detected by microscopic agglutination test (MAT). A confirmed case of leptospirosis was defined as having consistent clinical signs and any one of the following: seroconversion or four-fold increase in MAT titre for paired serum samples, or a MAT titre ≥ 1:400 for acute-phase serum samples. Twenty-nine of the 54 patients or convalescents tested for leptospirosis were seropositive. Two cases showed a confirmed infection for both plague and leptospirosis. While evidence supports the plague nature of this outbreak, the results suggest that some of the suspected plague cases might be due to leptospirosis. In any case, this diagnosis will have to be evoked in the future if a similar outbreak occurs in this region of Africa.

## 1. Introduction

Human leptospirosis is an emerging disease with over 500,000 severe cases occurring annually and a case fatality rate exceeding 10% [1]. Leptospirosis is a zoonosis caused by spirochetes of the genus *Leptospira*, bacteria that have a variety of mammalian hosts, particularly rodents. The bacterium enters the bloodstream via abrasions in the skin or through mucous membranes after contact with contaminated moist soil, water, or aerosolized droplets. Often, environmental contamination arises when leptospires are shed in urine of infected animal reservoirs such as rodents, cattle, pigs, and various wild animals. Leptospirosis is also transmitted to humans by direct contact with tissue or urine from infected animals. Human-to-human transmission is rare [2]. Severity of leptospirosis ranges from subclinical infection to severe forms including cardiac, renal, and pulmonary failures. Pulmonary findings for patients with leptospirosis were reported in 17%–70% of patients in several large studies and included hemoptysis, respiratory distress, cough, difficulty breathing, and pulmonary hemorrhage [3]. Pulmonary hemorrhagic syndrome caused by leptospirosis may have a mortality rate of up to 50% if left untreated [4]. 

Plague is a zoonosis caused by the bacterium *Yersinia pestis*, which circulates among small mammals and fleas. Bubonic plague is the most common form of the disease and may progress into a highly contagious pneumonic form, which can then be spread from person-to-person. The rapid onset and high lethality of pneumonic plague are its only distinguishable clinical features. The disease otherwise manifests itself as a severe respiratory infection that could be caused by various pathogens, such as the pulmonary form of leptospirosis [5]. 

In 2004, an outbreak suggestive of pneumonic plague erupted in a remote diamond mining camp in the Oriental Province of the Democratic Republic of the Congo (DRC), 25 km from the village of Zobia [6]. Diamonds were first discovered near Zobia in November 2004, and this attracted miners from the province and abroad. At the beginning of January 2005, approximately 7,000 people were living in the mining camp established in the area, located in the middle of the rain forest. The camp population included miners and traders and had extremely poor sanitary conditions. The “mine” consisted of pits dug in the ground, half-filled with stagnant water. The miners worked inside the pits, standing in the water for long periods of time. An outbreak of highly lethal pneumonia began on 15 December 2004, causing panic in the camp and surrounding area. By the beginning of March 2005, the population size had decreased to 2,500 after residents left due to fear of death brought on by the outbreak. The last case occurred on 11 March 2005. The outbreak resulted in 130 cases, including 57 deaths (44% fatality rate) [6].

A World Health Organization (WHO) team was tasked with the investigation and control of this outbreak. Due to a delayed alert and security constraints, the team arrived two months after onset of the epidemic. Forty-eight deaths had already occurred and no specimens had been taken. The number of case-patients available for clinical examination and appropriate sampling was, therefore, limited. Beyond plague, the presence of leptospirosis was investigated.

## 2. Experimental Section

Suspected deaths that occurred before the team’s arrival were attributed to plague on the basis of the epidemiologic context, reported clinical signs, and the rapid disease onset typical of pneumonic plague. Convalescents were tracked and interviewed. Additionally, blood samples were collected in standard serologic tubes by a clinician and stored at 4 °C for retrospective serologic testing. Each new patient with clinically suspected pneumonic plague was admitted to an isolation center. Blood was collected as previously described [6]. One serum sample was drawn as early as possible after onset of symptoms and a second serum sample was drawn 10 days later to allow for the development of an antibody response. Plague cases were classified according to the WHO classification [7]. Serum for leptospirosis investigation was transported at 4 °C or at ambient temperature and filter-sterilized upon arrival at the Institut Pasteur in Paris, France. Anti-*Leptospira* antibodies were detected by microscopic agglutination test (MAT) using the following antigens: serogroups Australis (serovar Australis), Autumnalis (serovar Autumnalis), Bataviae (serovar Bataviae), Canicola (serovar Canicola), Ballum (serovar Castellonis), Cynopteri (serovar Cynopteri), Grippotyphosa (serovar Grippotyphosa), Sejroe (serovars Hardjo and Sejroe), Hebdomadis (serovar Hebdomadis), Icterohaemorrhagiae (serovar Copenhageni), Panama (serovar Panama), Pomona (serovar Pomona), Pyrogenes (serovar Pyrogenes), Tarassovi (serovar Tarassovi), and Semaranga (serovar Patoc). The Semaranga serogroup belongs to a non-pathogenic leptospira species; it therefore cannot be an infecting serogroup. The serogroup was included in our analysis because it has cross-reactivity with pathogenic serogroups and can be indicative of an infection. Sera were screened at a dilution of 1/50 and positive sera were titrated to endpoint. High rates of agglutination of the serum with one particular antigen were used to identify the presumptive serogroup of the infecting bacterium.

## 3. Results and Discussion

### 3.1. Results

Fifty-four of the 82 patients observed after the response team’s arrival had sufficient serum quantities to allow for leptospirosis testing. Twenty-nine (53.7%) were seropositive for leptospirosis. Twenty had weak positive titres (<400), eight had a single strong positive titre (≥400), and one had a weak positive titre (<400) and seroconversion (Table 1). We were able to collect paired serum samples for six of the 54 patients tested for leptospirosis. Four of them were seropositive. One of these patients had a titre >400 for serogroup Canicola and was also a confirmed case of plague (Patient 3). We found one of these patients exhibited seroconversion for leptospirosis (Patient 27). Two convalescent samples were collected from Patient 2, one with a titre of 50 and the other one, which was negative, suggesting a previous infection, environmental exposure, or non-specific reactions. We also observed differences of reactivity to leptospiral antigens between the first and second specimen from the same individual (Patients 3 and 23), probably due to cross-reactions between serovars. 

The most frequently observed serological reactivity (MAT titre ≥ 100) was to serogroup Sejroe (12 patients), including seven subjects with high titre (titre ≥ 400). A significant seroreactivity (MAT titre ≥ 100) was also found for serogroups Canicola (five patients), Icterohaemorragiae (four patients), Bataviae (four patients), and Hebdomadis (two patients). For one patient, the MAT titres could not differentiate between serogroups Ballum and Sejroe. Other possible leptospirosis cases (nine patients) showed a low level of agglutination (titre 50) with reference serogroups (Table 1). 

**Table 1:** MAT results and identified serovars for 29 leptospirosis seropositive patients tested during the pneumonic plague outbreak, DRC, 2005.

Patient	Sex	Age	Patient v. Convalescent	Date of Symptom Debut	Specimen Collection Date	MAT	Leptospirosis Serogroups Detected (titre)	Classification
1	M	40	Convalescent	18-Feb-05	03-Mar-05	POS	Can (1:100)	P
2	M	42	Convalescent	30-Jan-05	26-Feb-05	POS	Bat (1:50) Can (1:50)	
2 (second specimen)	-	-	-	-	07-Mar-05	NEG	-	S
3	M	-	Convalescent	25-Jan-05	28-Feb-05	POS	Bat (1:50) Can (1:400) Cas (1:100) Ih (1:50)	
3 (second specimen)		-	-	-	07-Mar-05	POS	Bat (1:100) Cas (1:100) Ih (1:100)	C
4	M		Patient	27-Feb-05	02-Mar-05	POS	Sej (1:400) Pat (1:200)	C
5	M	34	Patient	22-Feb-05	27-Feb-05	POS	Bat (1:50)	S
6	F	-	Convalescent	18-Jan-05	27-Feb-05	POS	Gt (1:50) Sej (1:1600)	C
7	F	-	Patient	unk	02-Mar-05	POS	Bat (1:100)	P
8	M	28	Convalescent	unk	unk	POS	Sej (1:50)	S
9	M	-	Patient	unk	06-Mar-05	POS	Bat (1:50)	S
10	F	22	Patient	01-Mar-05	03-Mar-05	POS	Aus (1:100) Heb (1:100) Sej (1:400) Pat (1:50)	C
11	M	19	Patient	08-Mar-05	08-Mar-05	POS	Aus (1:50)	S
12	M	-	Convalescent	16-Feb-05	27-Feb-05	POS	Heb (1:100) Pat (1:50)	P
13	F	-	Convalescent	14-Feb-05	27-Feb-05	POS	Aus (1:50) Heb (1:50) Sej (1:1600) Pat (1:50)	C
14	M	32	Convalescent	21-Feb-05	08-Mar-05	POS	Can (1:200) Pat (1:100)	P
15	M	43	Convalescent	13-Feb-05	27-Feb-05	POS	Bat (1:50)	S
16	M	35	Convalescent	30-Jan-05	unk	POS	Can (1:50) Ih (1:100)	P
17	M	-	Patient	unk	08-Mar-05	POS	Can (1:200) Cas (1:50)	P
18	M	16	Convalescent	19-Jan-05	27-Feb-05	POS	Heb (1:50) Sej (1:1600)	C
19	M	38	Convalescent	04-Mar-05	10-Mar-05	POS	Ih (1:200) Sej (1:100)	P
20	F	-	Patient	unk	09-Mar-05	POS	Bat (1:50) Sej (1:50) Pat (1:50)	S
21	F	-	Convalescent	13-Feb-05	28-Feb-05	POS	Bat (1:50) Can (1:50) Cas (1:100) Sej (1:100) Pat (1:50)	P
22	M	-	Patient	unk	08-Mar-05	POS	Can (1:50) Sej (1:400)	C
23	F	47	Patient	03-Mar-05	4-Mar-05	POS	Cas (1:100) Sej (1:200) Pat (1:100)	
23 (second specimen)	F	47	Patient		09-Mar-05	POS	Bat (1:200)	P
24	F		Patient	unk	unk	POS	Sej (1:50)	S
25	M	26	Convalescent	05-Jan-05	8-Mar-05	POS	Sej (1:200)	P
26	F	46	Convalescent	06-Feb-05	8-Mar-05	POS	Sej (1:50)	S
27	M	23	Patient	01-Mar-05	03-Mar-05	NEG	-	
27 (second specimen)	-	-	-	-	09-Mar-05	POS	Bat (1:100) Can (1:100) Cas (1:100) Sej (1:200) Pat (1:50)	C
28	M		Patient	unk	27-Feb-05	POS	Aus (1:50) Ih (1:100) Pat (1:50)	P
29	F	60	Convalescent	04-Feb-05	2-Mar-05	POS	Aus (1:100) Gt (1:100) Sej (1:400)	C

Notes: Patient = specimen collected < 7 days of symptom onset, Convalescent = specimen collected ≥ 7 days of symptom onset. C = Confirmed, P = Probable, S = Suspect, unk = Unknown. Serogroups: Aus = Australis, Bat = Bataviae, Can = Canicola, Cas = Ballum, Gt = Grippotyphosa, Sej = Sejroe, Heb = Hebdomadis, Ih = Icterohaemorrhagiae, and Pat = Semaranga.

Within the group of eight patients having a strong positive result for leptospirosis, one was also confirmed (Patient 3) and one was probable for plague (Patient 13) (Table 2).

**Table 2:** Etiology of 29 leptospirosis seropositive patients tested during the pneumonic plague outbreak, DRC, 2005.

Patient	Sex	Age	Leptospirosis Classification	Plague Classification	Most Probable Etiology
1	M	40	P	S	Leptospirosis
2	M	42	S	C	Plague
3	M	-	C	C	Leptospirosis, Plague
4	M	-	C	S	Leptospirosis
5	M	34	S	S	und.
6	F	-	C	S	Leptospirosis
7	F	-	P	P	Leptospirosis, Plague
8	M	28	S	S	und.
9	M	-	S	S	und.
10	F	22	C	S	Leptospirosis
11	M	19	S	S	und.
12	M	-	P	S	Leptospirosis
13	F	-	C	P	Leptospirosis
14	M	32	P	S	Leptospirosis
15	M	43	S	S	und.
16	M	35	P	S	Leptospirosis
17	M	-	P	S	Leptospirosis
18	M	16	C	S	Leptospirosis
19	M	38	P	S	Leptospirosis
20	F	-	S	S	und.
21	F	-	P	S	Leptospirosis
22	M	-	C	S	Leptospirosis
23	F	47	P	C	Plague
24	F	-	S	S	und.
25	M	26	P	S	Leptospirosis
26	F	46	S	S	und.
27	M	23	C	C	Leptospirosis, Plague
28	M	-	P	S	Leptospirosis
29	F	60	C	S	Leptospirosis

Notes: C = Confirmed, P = Probable, S = Suspect, und = undetermined.

### 3.2. Discussion

It is estimated that Africa has the largest worldwide leptospirosis burden, with the highest median annual incidence of laboratory-confirmed cases (95.45 per 100,000 population) as well as the highest median annual mortality rate (5.5 per 100,000 population) but data, specifically in Central Africa, are scarce [4]. Leptospirosis in the DRC was investigated in the colonial period and three different foci were described in mining areas [8,9]. In Central Africa, miners are particularly exposed to infectious diseases due to the climate as well as very poor living and working conditions. Severe outbreaks are regularly reported, including cholera, epidemic meningitis, plague, and viral hemorrhagic fevers. The high prevalence of leptospirosis among workers on gold-bearing sites in the Belgian Congo was emphasized by Van Riel in 1946 [8]. Leptospirosis had also been detected and evoked as a differential diagnosis of Ebola fever in similar conditions in Gabon where a seroprevalence of 15.7% had been measured in the general population of remote villages, but the circulating leptospirosis serogroups had not been identified [10]. In Zobia, DRC, 29 of the 54 tested individuals were seropositive but were either patients or convalescents and cannot be considered representative of the whole population. Extrapolation to the general population is not even possible as the proportion of severe forms of leptospirosis is not clearly defined [2]. This finding, however, is in favor of an intense transmission of leptospirosis in the north-eastern part of DRC.

By MAT, the predominant serogroup was Sejroe (12/21 subjects with MAT titre ≥ 100), including three subjects with titre of 1,600, indicative of a recent infection. In 1954, the most prevalent *Leptospira* serogroups in Belgian Congo were Sejroe, Grippotyphosa, Hebdomadis, and Icterohaemorrhagiae. The identification of several other serogoups by MAT could be related to the multiple potential environmental sources of infection in the area of Zobia, as well as to the movement of infected people from neighboring regions and countries. It is also important to consider differences and advancements in testing assays, technology, and methodology between serogroup identification in 1954 and 2005. 

Identification of the serogroups may assist in determining animal reservoirs for leptospirosis, since a limited number of serovars can infect a given host [11]. Serogroup Sejroe was identified in many serological surveys in livestock worldwide [12,13,14,15]. This includes a study in Zimbabwe where Sejroe was found to be the predominant serogroup in beef cattle [14]. Knowledge of the serovars in this region will aid in improving diagnostic tools, identifying animal reservoirs, and generating control strategies. It is important to note that the accuracy of the MAT in predicting the infecting serovar may not be optimal, as the MAT panel strains did not contain local isolates [16,17].

The existence of a pneumonic plague outbreak cannot be denied; five cases were confirmed and ten were considered as probable according to the WHO case definition (Table 2) [6]. In a region where plague had never been reported, this is an event of international public health importance by itself. However, the possibility of other etiologies able to mimic pneumonic plague was considered early in the investigation. One cannot disregard the possibility that a certain number of the 130 reported cases were not due to plague nor plague alone. When considering the role of leptospirosis in this outbreak it must first be noted that not all leptospirosis infection cause clinical disease. In addition, it was impossible to confirm the etiology of each of the reported cases due to the field conditions, delayed investigation, and unmonitored use of antibiotics. Of the 82 patients and convalescents observed after the response team’s arrival, only 54 could be tested for leptospirosis in addition to plague. Discussion about the respective contributions of the two etiologies is therefore restricted to 41.5% of the severe cases of pneumonia diagnosed during this outbreak. With these limitations in mind, we can reasonably consider the etiology as solely plague for two patients (2 and 23) and solely leptospirosis for two other patients (6 and 18). Patient 27, who seroconverted for leptospirosis but was also confirmed for plague, suggests that such a co-infection is possible. Clinically, leptospirosis in its pneumonic presentation can mimic plague. Plague, however, usually leads to death within 2–4 days after exposure, which is not the case for leptospirosis [18]. Specific details of clinical presentation were not recorded and conditions of hospitalization prevented complementary investigations, however, these patients recovered. Like all the patients managed during the outbreak response, they were treated with gentamicin. While gentamicin is not the currently recommended antibiotic therapy for leptospirosis, this antibiotic could be effective on pathogenic *Leptospira* [19]. The existence of leptospirosis cases among the victims could partly explain the relatively low case fatality ratio (11.0%) observed during the field investigation [6].

Leptospirosis is usually considered in instances of febrile jaundice, even though jaundice is not the most common clinical manifestation [2]. This makes leptospirosis a differential diagnosis of yellow fever in central Africa. Two cases of yellow fever were confirmed in 2010, 70 km north of Zobia [20]. Similarly, a haemorrhagic syndrome could be the manifestation of either leptospirosis or a haemorrhagic viral fever [10]. Due to its potential high mortality and pulmonary manifestations consistent with those of pneumonic plague, leptospirosis should be evaluated as a differential diagnosis for pneumonic plague within the region and for similar outbreaks in similar conditions in order to provide effective treatment and implement appropriate public health measures to slow, stop, and prevent disease spread.

It is unclear yet what the potential is for specific leptospirosis outbreaks in this region. More needs to be done to understand the importance of leptospirosis and its natural history and disease burden in central Africa.

The complexity of leptospirosis laboratory confirmation makes early diagnosis difficult in developing countries. A new generation of rapid tests, more reliable and usable in the field than the current ones, is needed. Until such tests are available and in use, regional health staff should be educated on leptospirosis presentation and first-line diagnosis capacities should be reinforced.

The investigation was impacted by hectic working conditions during a plague outbreak in which resources were limited and activities focused on the immediate task of saving lives. This resulted in several research limitations. Specific details of clinical presentation were not recorded and conditions of hospitalization prevented complementary investigations. Moreover, it was not possible to compare the observed seroprevalence with that of a reference population, which restricts the interpretation. Our findings, however, are valuable as very little is known about leptospirosis in Africa.

## 4. Conclusions

While plague remains the main culpable agent for the outbreak of severe pneumonia in the miners' camp, a leptospirosis outbreak co-existed. This is the first time that leptospirosis has been described in DRC since 1956. The circulation of *Leptospira* is likely intense in the natural environment of the north-eastern part of the country, especially around mining activity areas. Leptospirosis may be accounting for at least a small portion of illnesses in other severe outbreaks occurring in central Africa. Due to its potential high mortality and pulmonary manifestations consistent with those of pneumonic plague, leptospirosis should be evaluated as a differential diagnosis for pneumonic plague within the region and for similar outbreaks in similar conditions in order to provide effective treatment and implement appropriate public health measures to slow, stop, and prevent disease spread.
